# A Randomized Crossover Trial on Acute Stress-Related Physiological Responses to Mountain Hiking

**DOI:** 10.3390/ijerph14080905

**Published:** 2017-08-11

**Authors:** Martin Niedermeier, Carina Grafetstätter, Arnulf Hartl, Martin Kopp

**Affiliations:** 1 Department of Sport Science, University of Innsbruck, Fürstenweg 185, 6020 Innsbruck, Austria; martin.kopp@uibk.ac.at; 2 Institute of Ecomedicine, Paracelsus Medical University, Strubergasse 22, 5020 Salzburg, Austria; c.grafetstaetter@pmu.ac.at (C.G.); Arnulf.Hartl@pmu.ac.at (A.H.)

**Keywords:** green exercise, urbanization, cortisol, heart rate variability, blood pressure, allostatic load

## Abstract

Green exercise, defined as physical activity in natural environments, might have positive effects on stress-related physiological measures. Little is known about the acute effects of green exercise bouts lasting longer than 60 min. Therefore, the aim of the present study was to analyze the acute effects of a three-hour green exercise intervention (mountain hiking) on stress-related physiological responses. Using a randomized crossover design, 42 healthy participants were exposed to three different conditions in a field-based experiment: outdoor mountain hiking, indoor treadmill walking, and sedentary control condition (three hours each). At baseline and at follow-up (five minutes after the condition), stress-related physiological responses (salivary cortisol, blood pressure, and heart rate variability) were measured. Salivary cortisol decreased in all conditions, but showed a larger decrease after both mountain hiking and treadmill walking compared to the sedentary control situation (partial η^2^ = 0.10). No differences were found between mountain hiking and treadmill walking in salivary cortisol. In heart rate variability and blood pressure, changes from baseline to follow-up did not significantly differ between the three conditions. The results indicate that three hours of hiking indoors or outdoors elicits positive effects on salivary cortisol concentration. Environmental effects seem to play a minor role in salivary cortisol, blood pressure, and heart rate variability.

## 1. Introduction

Urbanization, considered as an increase in population in urban regions versus a decrease in population in rural areas, is an ongoing process worldwide. Since 2008, more than 50% of the global population has been living in urban areas [1]. Urbanization is connected to physical, social, behavioral, and economic changes for the population, e.g., increased pollution, sedentary lifestyle, stress and stress-related diseases [2,3]. These changes represent a large challenge for the public health system, e.g., sedentary lifestyle is connected with increased mortality [4] and high economic costs [5]. Stress and insufficient recovery from stress increase allostatic load, which is defined as the “wear and tear on the body and brain resulting from chronic overactivity or inactivity of physiological systems that are normally involved in adaptation” [6], has long-term effects on health and is considered as a risk factor for Western lifestyle diseases such as cardiovascular diseases, diabetes and mental disorders [7]. Therefore, interventions to reduce stress and measures to support recovery from stress are highly needed. 

There is evidence that exposure to natural environment (without physical activity) shows positive effects on stress reduction, restoration and physical recovery from surgery [8,9]. Furthermore, physical activity (without exposure to natural environment) showed similar effects on stress reduction [10,11]. Physical activity and exposure to natural environments can be combined (often referred to as green exercise) and may have synergistic effects on stress-related psychological and physiological measures. For psychological measures, there is meta-analytic evidence that self-esteem and affective responses are improved with a medium-sized effect from pre- to post-green exercise [12]. However, the calculation of the effect sizes reported were based on the values before and after green exercise and were not compared with exercise in other areas such as indoor or urban environments. Compared to indoor exercise, increasing feelings of revitalization and increased energy after green exercise have been reported in the meta-analysis of Thompson Coon, et al. [13]. Also compared to both indoor and synthetic environments, positive effects on psychological variables on the basis of (systematic) reviews have been reported [14,15]. In a stress-related context, a positive impact on affective responses is desirable for at least two reasons. First, negative affective state is connected with higher stress level measured by cortisol concentration [16]. Second, positive affective responses during physical activity may increase future physical activity behavior [17,18,19], which is known to have positive long-term effects on stress-related diseases.

Aside from the impact of green exercise on psychological variables, there is growing interest in its acute effect on stress-related physiological measures. Physiological measures include cardiovascular (e.g., blood pressure, heart rate variability), endocrine (e.g., adrenaline, noradrenaline, cortisol), immune function (e.g., natural killer cells, immunoglobulin A), and brain activity measures measured by electroencephalography [15]. The effect on physiological measures seems to be less consistent compared to psychological measures. A laboratory study showed positive effects on blood pressure while exercising in front of photographs of rural environments compared to photographs of urban environments [20]. By contrast, Bowler, Buyung-Ali, Knight and Pullin [14] summarized 25 studies on walking or running in green exercise. Compared to indoor/synthetic environments, the authors could not find additional positive effects of green exercise on blood pressure or cortisol levels. Haluza, Schonbauer and Cervinka [15] reported mixed results in cardiovascular and endocrine measures. Four out of nine studies reported larger positive impact of green exercise compared to indoor/synthetic outdoor exercise. The authors mentioned several limitations of the studies: (a) low statistical power due to small sample sizes; (b) mainly Japanese studies, where anticipation/expectation effects might play a larger role compared to European studies; (c) predominantly male participants; and (d) cross-sectional design/design without control intervention. Furthermore, the type of intervention (e.g., intensity, duration) might influence the results. Regarding the duration of green exercise, the majority of studies used exercise interventions of up to 60 min duration [15]. Little is known about the effects of green exercise bouts of a duration longer than 60 min.

Mountain hiking, considered as walking in green, mountainous areas with altitude differences, can be considered as an exercise that is typically of longer duration. Despite the fact that the duration of mountain hiking tours shows a large variation up to a whole day, tours with a mean duration of approximately three hours were reported previously [21,22]. Consequently, mountain hiking can be considered as an appropriate example to study effects of longer-lasting physical activity bouts. 

Thus, the aims of the present study were to analyze acute effects of (a) the environment and (b) longer-lasting physical activity on stress-related physiological parameters. We compared green exercise (mountain hiking, approximately 3 h) with indoor exercise and with a sedentary control situation of an identical duration. In the context of the literature, we hypothesized that the exercise conditions might show more favorable effects compared to the sedentary condition and that the environment of the green exercise condition might have additional favorable effects compared to indoor exercise. 

## 2. Materials and Methods

### 2.1. Design and Procedure

**Figure 1:**
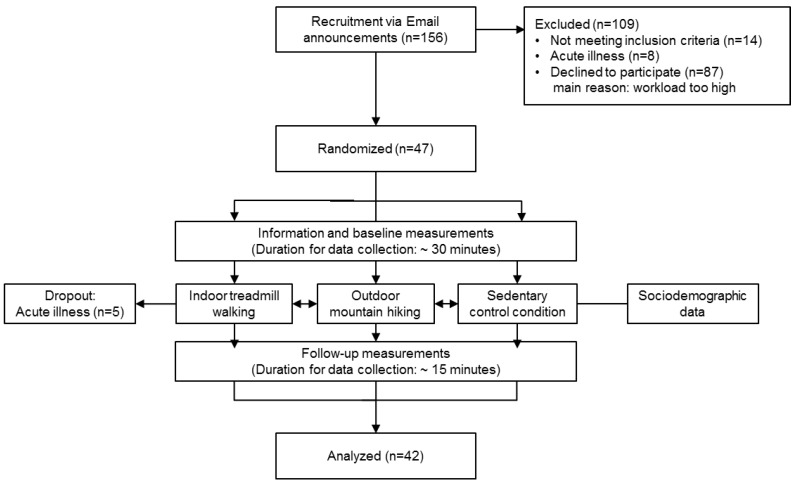
Flow diagram for data collection and participant flow. All participants were exposed to the three experimental conditions in a randomized order. The cases of acute illness occurred after the sedentary control condition (n = 2) and after indoor treadmill walking (n = 3).

The present study was embedded in a larger study previously published [23]. Briefly, all participants were exposed to three experimental conditions in a randomized order: outdoor mountain hiking, indoor treadmill walking, and sedentary control condition. After baseline measurements, the three-hour intervention started (outdoor mountain hiking, indoor treadmill walking, or sedentary control condition), see Figure 1. Upon completing the follow-up measurements with identical measurements of the baseline, the participants were instructed for the next condition and departed individually. All measurements were performed in the group in a sedentary position and were supervised by one researcher (MN). The participants were in a sedentary position for five minutes before physiological measures were taken in the following order: blood pressure, salivary cortisol concentration, and heart rate variability. The order of the measurements, the timeline and the time of day remained identical for all three experimental conditions. The mean washout phase between the conditions was planned to be one week; however, due to conflicting schedules, the washout phase varied from one to 14 days. One researcher (MN) supervised and took part in all conditions.

The study was approved by the Institutional Review Board of the Department of Sport Science of the University of Innsbruck (22 April 2015) and all participants signed a consent form after obtaining written and spoken information about the study procedures. The trial was registered at ClinicalTrials.gov (identification number: NCT02853760, retrospectively registered).

### 2.2. Participants

Participants were healthy adults living in Innsbruck and were recruited by public and by Email announcements sent by the University of Innsbruck. Exclusion criteria were: (a) pregnancy; (b) breast-feeding; (c) chronic or acute diseases (already existing or diagnosed during the study); (d) age below 18 and above 70 years; (e) unable to be physically active assessed by the Physical Activity Readiness Questionnaire [24]. No incentive was provided for participation in the study.

An a priori power analysis was performed to estimate the appropriate sample size. Using G*Power 3.1 [25], a sample size of n = 45 participants was calculated for detecting a significant condition by time interaction using a three × two fully repeated measures ANOVA. The following assumptions were used: α = 0.05, power = 0.80, dropout rate: 20%, partial η^2^ = 0.07 based on previous research [26].

### 2.3. Interventions

Outdoor mountain hiking was conducted in a famous mountain hiking area in Innsbruck. After information and baseline measurements outdoors sitting on a bench (900 m), participants hiked uphill for 6 km on single trails and forest roads to a mountain hut (1500 m) with a view of the mountainous region. The participants were instructed to choose an intensity corresponding to “brisk without overspending” pace (average speed uphill: 4 km/h). After a resting phase in a sedentary position for 10 min, the participants were hiking downhill on the same track to the starting point to the follow-up measurements (average speed downhill: 5.2 km/h). Uphill hiking phase lasted approximately 90 min and downhill hiking phase around 70 min.

Indoor treadmill walking was conducted in a fitness center in Innsbruck (590 m). After recording the baseline values, all participants were walking uphill on treadmills for the first part of the intervention. The following settings were adjusted on the treadmill in the uphill situation: inclination: 10%, time: 1.5 h, and speed: 4 km/h (resulting in 600 m difference in altitude). In accordance to possible differences in outdoor speed, the participants were allowed to change the treadmill’s speed in a small range (3.8 to 4.2 km/h) to adapt to the wording “brisk without overspending”. After resting for 10 min, the second part of the intervention contained 70 min of level walking on the same treadmills (5.2 km/h, 6 km). Unfortunately, downhill walking was not possible on the treadmills used. 

The sedentary control condition was located in a quiet room at the University of Innsbruck with access to computers. The participants were allowed to use the computers, to read, and to talk, but had to remain in a sedentary position (short pauses for using the restroom were allowed). Sociodemographic data (age, sex, height, weight, physical activity, mountain sport experience) were collected for 5 to 10 min at the beginning of the sedentary control condition using a web-based questionnaire.

### 2.4. Measurements

Endocrine and cardiovascular physiological measures were used as markers of stress and sympathetic-parasympathetic activity: salivary cortisol concentration, heart rate variability and blood pressure.

Salivary cortisol concentration: Salivary samples (2.5–3.5 mL) were collected in polypropylene vials using an unstimulated passive drooling method. All samples were stored separately at −20° until cortisol concentrations were determined collectively at the laboratory of the Paracelsus Medical University, Salzburg, Austria. After thawing and reaching room temperature, the samples were centrifuged at 1500 × *g* for 10 min to separate mucins. Samples were analyzed using a Cortisol Saliva ELISA Free (SA E-6000) assay from LDN^®^ (Labor Diagnostika Nord GmbH & Co. KG, Nordhorn, Germany) according to manufacturer’s instructions. The absorbance of each sample got determined at 450 ± 10 nm with a calibrated microtiter plate reader (Anthos Zenyth 3100, model DTX 880, Friesoythe, Germany). The observed intra- and inter-assay coefficients of variation were 2.1 to 3.1% and 4.1 to 4.4%, respectively. Means of the reported intra- and inter-assay coefficients of variation were 4.8% and 6.3%, respectively. Concentrations were calculated using a four-parameter logistics (4 PL) curve fit and expressed in nmol/L. Higher cortisol concentrations indicate higher levels of allostatic load.

For cardiovascular parameters, heart rate variability (HRV) and blood pressure (BP) were assessed. Systolic and diastolic BP was collected using an oscillometric technique device (M4 Plus; Omron, Germany). BP was measured at the brachial artery of the left arm (single measurement). HRV data were collected for 5 min in a sitting position and participants were asked not to talk or move. A wireless heart rate transmitter (sampling rate: 1000 Hz, 2-lead electrocardiography) placed on a belt around the chest and a wristwatch (RS800CX, Polar Electro Oy, Espoo, Finland) were used to obtain RR intervals. Excellent reliability (intraclass correlation coefficient > 0.9) and validity values have been shown previously, especially for resting phases [27]. For HRV data processing, integrated Polar software (Polar Pro Trainer) was used. Polar software to analyze HRV data showed results with adequate precision [28]. After artefact correction, HRV indices of time and frequency domain were calculated on the basis of RR intervals according to the guidelines of the Task Force of The European Society of Cardiology [29].

Time domain indices contained the standard deviation of the normal-to-normal beat intervals (SDNN [ms]), the square root of the mean squared differences of successive normal-to-normal beat intervals (RMSSD [ms]). SDNN reflects all the cyclic components responsible for variability and RMSSD reflects high frequency variations [29,30]. Lower SDNN/RMSSD values indicate lower HRV and were considered as higher levels of stress.

Frequency domain indices contained power in low frequency range (LF [ms^2^]) in the spectrum of 0.04–0.15 Hz and power in high frequency range (HF [ms^2^]) in the spectrum of 0.15–0.40 Hz. Furthermore, total power [ms^2^], normalized LF (LFn), normalized HF (HFn), and the ratio between LF and HF (LF/HF [%]) were calculated. Although parasympathetic and sympathetic activities are closely connected, LF and LFn are believed to mainly reflect sympathetic activity and HF and HFn mainly parasympathetic activity. LFn, HFn and LF/HF reflect the sympathetic-parasympathetic balance and are considered to indicate comparable aspects of HRV [31].

Walking intensity was measured by heart rate (HR) as an objective measure and self-reported perceived exertion as a subjective measure in outdoor mountain hiking and indoor treadmill walking. HR was continuously measured throughout the intervention time between baseline and follow-up using a heart rate transmitter and polar watch (RS800CX, Polar Electro Oy, Espoo, Finland). Values of the resting phase were excluded from the analysis. Estimated percentage of maximal heart rate was calculated on the basis of mean HR values over the intervention time using the formula of Tanaka, et al. [32]. Self-reported Rating of Perceived Exertion (RPE [33]) was assessed at the end of uphill walking and at the end of level walking (indoor treadmill walking)/downhill walking (outdoor mountain hiking) retrospectively with respect to the corresponding intervention time. The RPE ranges between 6 (“no exertion”) and 20 (“maximum exertion”) and is considered a valid assessment method of perceived exertion [33,34].

Salivary cortisol concentration was the main outcome of the study. Secondary outcomes were heart rate variability, blood pressure, heart rate and rating of perceived exertion during the interventions.

### 2.5. Statistical Analyses

All statistical analyses were performed using SPSS version 23 (IBM, New York, NY, USA). Possible differences at baseline were tested by separate one-factorial analyses of variances with repeated measurements (ANOVAs) with condition as within-factor (outdoor mountain hiking, indoor treadmill walking, sedentary control condition). 

For each of the outcome measures, a three × two fully repeated measures ANOVA was used to analyze the effect of condition (outdoor mountain hiking, indoor treadmill walking, sedentary control condition), time (baseline, follow-up) and condition by time interactions. Significant interactions between condition and time were considered as different changes in the parameters. When significant interactions were found, pre-planned simple contrasts were used with outdoor mountain hiking as reference category to analyze effects due to exercise (compared to sedentary control condition) and effects due to the environment (compared to indoor treadmill walking) [35]. In salivary cortisol concentrations, we also contrasted indoor treadmill walking vs. sedentary control condition.

Additional to the main analysis, the influence of the group factors sex and blood pressure was analyzed. A series of mixed model ANOVAs with the repeated measures factors condition and time and the group factors sex (female, male) and blood pressure group (normotensive, (pre)hypertensive). Normotensive was defined as systolic blood pressure 120 mmHg and below during baseline at the sedentary control condition and (pre)hypertensive was defined as systolic blood pressure above 120 mmHg. Pre-hypertensive and hypertensive subjects were combined, since only four hypertensive subjects participated (i.e., systolic blood pressure > 140 mmHg). The interaction of condition by time by sex and the interaction of condition by time by blood pressure group were inspected to answer the question if sex or blood pressure group showed a significant influence on the condition by time interaction.

Four cortisol values at baseline could not be analyzed (missing values: 1.6%) and were replaced by the respective sample mean of the condition following the approach of Liu, et al. [36]. Since cortisol and frequency domain indices of HRV measures were not normally distributed, transformation of the values was conducted. Cortisol concentrations were square root-transformed and frequency domain indices of HRV were log-transformed before the analysis. Whenever the assumption of sphericity was not met in the ANOVA, Greenhouse-Geisser correction was applied.

The significance level was set at α = 0.05 (two-tailed). Since multiple outcome measures were analyzed, Bonferroni correction was applied and resulted in *p*-values of 0.025 (HRV time domain indices), 0.008 (HRV frequency domain indices), and 0.025 (BP). Partial η squared was used as an effect size with the classifications small (0.01), medium (0.06), and large (0.14) [37]. Unless otherwise stated, data is presented as mean (standard deviation).

## 3. Results

Table 1 shows the demographic data of the 42 participants (48% female, 59% membership in an alpine association, 38% married/de facto partnership, 60% university degree). Significant differences at baseline were found for systolic BP, F(2,80) = 6.20, *p* = 0.003, and diastolic BP, F(1.6,65.0) = 7.20, *p* = 0.003. At baseline, both systolic and diastolic BP were higher before outdoor mountain hiking compared to sedentary control condition and indoor treadmill walking. No differences at baseline between the conditions were found for any other parameters (all *p* > 0.05). No harmful event to the participants was observed in any of the conditions.

### 3.1. Salivary Cortisol Concentration and Cardiovascular Parameters

**Figure 2:**
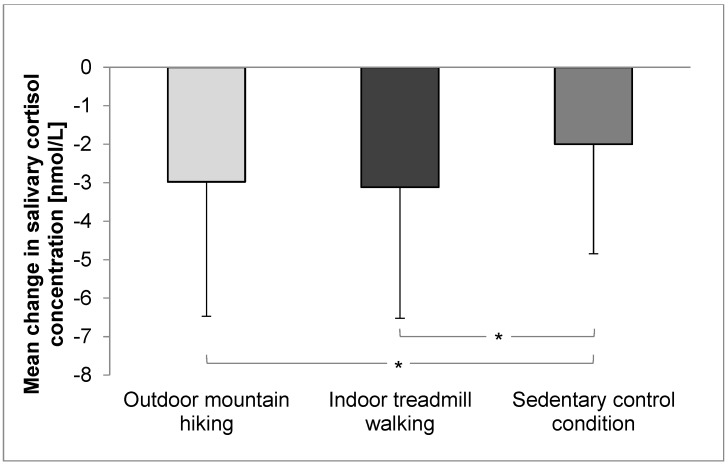
Mean changes in salivary cortisol concentration from baseline to follow-up by condition. * Significant condition by time interaction, error bars represent standard deviations.

For salivary cortisol concentration, there was a significant time effect indicating a decrease over time in all conditions (Table 2). Additionally, a significant interaction between time and condition was evident. The decrease in cortisol concentration was larger after outdoor mountain hiking compared to sedentary control condition and larger after indoor treadmill walking compared to sedentary control condition (Figure 2). There was no significant interaction contrast between outdoor mountain hiking and indoor treadmill walking indicating comparable decreases in cortisol concentration after outdoor mountain hiking and indoor treadmill walking. 

In both time domain indices of HRV (SDNN and RMSSD), a significant increase from baseline to follow-up was found, but no significant condition by time interaction. None of the frequency domain indices of HRV showed significant interaction effects indicating comparable changes over all three conditions. Only total power showed a significant increase over time in all three conditions. 

Both systolic and diastolic BP showed a significant condition effect. Simple contrasts revealed a significantly higher systolic and diastolic BP in outdoor mountain hiking compared to indoor treadmill walking and sedentary control condition. Additionally, a significant time effect for systolic BP was found. Systolic BP was higher at baseline compared to follow-up in all three conditions. No significant interaction effect was found for systolic and diastolic BP indicating similar changes in all three conditions. 

### 3.2. Influence of Sex and Blood Pressure on Salivary Cortisol Concentration and Cardiovascular Parameters

No significant results were found in the condition by time by sex interaction, *p* > 0.045, and in the condition by time by blood pressure group interaction, *p* > 0.210, indicating a similar condition by time interaction over the factors sex and blood pressure group.

### 3.3. Walking Intensity 

Mean heart rate was slightly higher in outdoor mountain hiking with 111 (17) bpm compared to indoor treadmill walking with 105 (14) bpm. When expressed as percentage of estimated maximal heart rate, participants were walking in outdoor mountain hiking with 60.0 (10.0)% and in indoor treadmill walking with 56.7 (8.1)% of the maximal heart rate. Mean RPE was slightly lower in outdoor mountain hiking with 10.4 (1.6) compared to indoor treadmill walking with 10.9 (2.1). 

## 4. Discussion

### 4.1. Main Results

The results of the present study suggest that a single three-hour bout of mountain hiking (both indoors and outdoors) elicits a stronger reduction of salivary cortisol levels compared to a sedentary control situation. No such effect was found for blood pressure and heart rate variability. We could not detect additional positive effects on stress-related physiological parameters salivary cortisol level, blood pressure, and heart rate variability due to the natural environment. 

### 4.2. Effects of Physical Activity on Stress-Related Physiological Parameters 

The difference between physical activity with moderate intensity and a sedentary condition is in line with previously reported results in healthy and clinical populations [38,39]. Consequently, exercise with moderate intensity might be considered as an intervention with stress-buffering effects and might be recommended as a prevention against cardiovascular diseases, diabetes and mental disorders [7]. Our data add to the research field positive effects of longer lasting (i.e., three hours) exercise interventions. Until now, research on exercise bouts shorter than 60 min was predominantly available. It is important to provide evidence for forms of exercise which are regularly conducted by the population. In 2005, nearly 45% of the Austrian population above the age of 15 years was practicing various forms of mountain exercise [40,41]. This number is supplemented by more than 40 million tourists, who are regularly visiting mountainous regions in the Alps [42]. Aside from exercise duration, exercise intensity seems to play an important role, since vigorous exercise (i.e., exercise above 60% of the maximal oxygen uptake corresponding to ca. 70% of maximal heart rate) is considered as an acute stressor and can therefore increase cortisol concentrations after exercise [43,44].

We did not observe significant changes from baseline to follow-up between the conditions in HRV; neither in time domain nor frequency domain based indices of HRV. It might be concluded that sympathetic-parasympathetic activity cannot be influenced by a single three-hour bout of mountain hiking compared to a sedentary control condition. It should be noted that we applied a strict Bonferroni correction especially in the frequency domain indices (α corr = 0.008) following the approach of Lackner et al. [45]. However, we observed non-significant large-sized effects between the physically active conditions and sedentary control condition (Table 2). Possibly, the Bonferroni correction was too strict to reveal an increase of the sympathetic tone after mountain hiking and treadmill walking compared to the sedentary control condition. Previous uncontrolled studies reported increased LFn and decreased HFn after exercise [46,47]. The time point of measurement of HRV after exercise seems to be crucial. Since we measured HRV at a maximum of 10 min after exercise, the increase of the sympathetic tone might be a direct effect of the exercise. It can be expected that this increase of sympathetic tone decreases after recovery [46]. Compared to cortisol concentration, which is affected after a time delay (i.e., a minimum of 15 min, Kirschbaum and Hellhammer [48]), HRV seems to mirror the acute activation of sympathetic tone due to physical activity.

Congruent with existing literature, we expected a decrease in both systolic and diastolic BP after exercise compared to the control condition. This effect is known as post exercise hypotension [49]. Although a significant decrease for systolic BP after exercise was present, there was no larger decrease after the exercise conditions compared to the sedentary control condition. Although the mean difference of systolic BP in mountain hiking was 5.9 mmHg compared to 1.8 mmHg in the sedentary control condition, the significance threshold was missed. Since BP can be considered a relatively sensitive measure (similar to HRV), we question whether BP is an appropriate parameter in field experiments with longer-lasting exercise conditions.

### 4.3. Environmental Effects on Stress-Related Physiological Parameters

We could not detect a different change from baseline to follow-up between mountain hiking and treadmill walking. This was observed for cortisol concentration, heart rate variability and blood pressure. It might be concluded that exercising in a green environment does not have an additional benefit in stress-related physiological measures compared to an indoor environment. 

This is in line with some, but not with other studies summarized in Haluza, Schonbauer and Cervinka [15] and Barton and Pretty [12]. Explanations for these mixed results might be found in various reasons: First, especially HRV and BP can be considered as parameters sensitive to external perturbations (e.g., noise, sun exposure, and breathing). Compared to a laboratory study, these influences can hardly be controlled in a field study, which compounds the ability to detect environmental influences [50]. Second, circadian fluctuations might play a larger role in three hours interventions compared to shorter interventions. Therefore, the circadian effect might have superimposed possible intervention effects. Third, the majority of studies with positive results on stress-related parameters were conducted in Japan, where Shinrin-Yoku, a special form of green exercise, is considered a “recognized stress management activity” [51]. Consequently, participants might anticipate a positive effect already before being exposed to natural environments [15]. Fourth, in the present study, we used a controlled design with baseline measurements and corrections for multiple outcome measures, which was not the case in all previous studies. Positive green exercise effects were found predominantly in uncontrolled studies [12], studies without baseline measurements [52], or without appropriate adjustment for multiple comparisons [53].

Similar to the present study, more recently conducted studies with a shorter exercise duration using a controlled design could not find environmental differences on stress-related physiological parameters. Tyrväinen, et al. [54] used a similar study design and compared the effects of moderate walking exercise in an urban and park environment on salivary cortisol concentrations. The authors reported “no differences in cortisol levels” due to different environments. For HRV, Gidlow, Jones, Hurst, Masterson, Clark-Carter, Tarvainen, Smith and Nieuwenhuijsen [50] reported that “when taking measurements in the field, rather than in laboratory conditions, (…) [the] ability to detect subtle environmental responses using HRV” might be limited. Environmental effects might play a larger role in perceived psychological parameters (e.g., affective responses, mood) compared to physiological parameters [23,54]. 

### 4.4. Other Sources of Influence on Stress-Related Physiological Parameters 

We observed a significant decrease of cortisol concentrations from baseline to follow-up in all conditions, which can be explained by the circadian rhythm. Nater, et al. [55] reported a similar decrease for the corresponding time (approximately 3 to 7 p.m.). However, being moderately physically active for three hours resulted in a larger decrease of salivary cortisol concentrations. The increase in time domain indices of HRV might also be explained by the circadian rhythm [56]. The significant decrease in systolic BP might be partially mediated by a hypotensive effect of exercise [49].

Previous research has shown differences between male and female in stress-related physiological parameters [57,58]. However, concerning the acute effects of exercise or environmental effects, sex seems to have a minor influence according to the present results. A similar conclusion might be drawn for the influence of blood pressure. We did not observe that (pre)hypertensive individuals showed different change scores in any of the parameters between the conditions compared to normotensive individuals. Since high blood pressure is considered a risk factor for stress-related diseases and mortality [59,60], it would be desirable that especially individuals with high blood pressure benefit from interventions to reduce stress-related physiological parameters. However, it has to be noted that the conclusions of the influence of sex and blood pressure group are based on smaller subgroups.

### 4.5. Limitations

At least five limitations have to be considered when interpreting the findings: First, it was not feasible to apply downhill walking in the indoor situation, which resulted in a different form of physical activity during the second part of the intervention. It remains, therefore, unknown if possible differences between the environments were masked by the different form of physical activity. Downhill walking contains a higher percentage of eccentric muscle activity compared to level walking and was shown to cause different metabolic responses compared to uphill walking [61]. Consequently, also salivary cortisol response, blood pressure and heart rate variability might be affected differently. Second, the difference in mean heart rate could have influenced the results. Heart rate was significantly higher during mountain hiking compared to indoor walking. Since duration and speed in both exercise conditions were controlled, this discrepancy may have occurred by unequal surface and therefore a higher muscular activity in outdoor hiking or temperature differences between indoors and outdoors. Third, a possible selection bias cannot be ruled out in the present study. The recruitment was done on a voluntary basis without compensation despite the amount of around 12 h of workload. It might be assumed that participants who were already practicing (mountain) exercise were attracted by the study announcement. Since announcements were done at the University, we additionally faced a high rate of participants with University degree. Fourth, the length of the washout phase was not identical in all subjects. Although baseline measures were provided, it might be possible that longer-term effects of the previous condition influenced the change score in the following condition. Furthermore, the menstrual cycle was not considered in the washout phase in females, although cortisol levels are influenced by the menstrual cycle [57]. Future studies might consider controlling the washout phase for menstrual cycle in females. Fifth, we did not apply an acute stressor after the intervention to measure stress reactivity. Although it seems to be a common approach in green exercise studies to assess cortisol levels without acute stressor [15,50,54], future studies might consider measuring stress reactivity, i.e., cortisol responses to an experimental stressor (e.g., cognitive tasks or public speaking). Knowledge about the impact of (green) exercise on stress reactivity might provide additional insights about potential stress-buffering effects, which could be of high practical relevance in the field of behavioral stress management. Furthermore, future studies might consider measuring the general stress level of the participants at baseline.

## 5. Conclusions

The findings of the present study indicate that a single bout of three-hour green exercise intervention (mountain hiking) elicits positive effects on salivary cortisol concentration compared to a sedentary control condition. Thereby, findings on effects of shorter bouts of exercise with moderate intensity were confirmed. However, the effects on physiological responses during mountain hiking seem to be caused by the physical activity and not by environmental effects, since no differences were found between mountain hiking and treadmill walking. Environmental effects might play a larger role in psychological parameters compared to stress-related physiological parameters. 

## Figures and Tables

**Table 1:** Demographic data of the study participants for the total group and by sex.

	Total Group			Female	Male
	Mean (SD ^1^)	Minimum	Maximum	Mean (SD ^1^)	Mean (SD ^1^)
Age (years)	32.0 (12.0)	19.0	66.0	35.9 (15.7)	28.5 (7.3)
Height (m)	1.74 (0.10)	1.52	1.95	1.66 (0.08)	1.82 (0.06)
Weight (kg)	69.0 (11.0)	46.0	92.0	60.9 (6.7)	77.0 (7.7)
Body mass index (kg/m^2^)	23.0 (2.0)	18.1	26.6	22.1 (2.2)	23.3 (1.5)
Physical activity (h/week)	8.0 (5.0)	0.0	25.0	7.0 (3.9)	9.2 (6.3)
Mountain tours (n/year)	27.2 (26.2)	0.0	100.0	36.7 (27.0)	18.6 (22.7)

^1^ SD: Standard deviation.

**Table 2:** Mean (SD) values for cortisol concentration and cardiovascular parameters from baseline (BL) to follow-up (FU) by condition.

	Outdoor Mountain Hiking		Indoor Treadmill Walking		Sedentary Control Condition		*p*-Value			η^2^p ^1^	
	BL ^2^	FU ^3^	BL ^2^	FU ^3^	BL ^2^	FU ^3^	Condition	Time	Interaction	M-C ^4^	M-T ^5^
Cortisol [nmol/L]	4.7 (3.7)	1.8 (1.2)	5.0 (3.5)	1.8 (1.1)	4.3 (3.2)	2.3 (1.8)	0.616	<0.001 *	0.032 *	0.10	0.00
SDNN ^6^ [ms]	76.3 (40.9)	85.4 (47.5)	70.7 (40.6)	77.9 (41.6)	74.2 (51.3)	86.5 (32.4)	0.518	0.009 *	0.833	0.00	0.00
RMSSD ^7^ [ms]	58.4 (44.5)	68.6 (56.6)	51.5 (43.9)	57.3 (41.0)	46.7 (27.0)	61.8 (27.7)	0.402	<0.001 *	0.502	0.02	0.00
Total power [ms^2^]	9497 (10,715)	10,504 (10,254)	7906 (10,552)	10,055 (10,574)	10,443 (26,532)	11,568 (9456)	0.127	0.005 *	0.030	0.12	0.01
LF ^8^ [ms^2^]	2331 (2399)	2967 (2729)	1973 (2934)	2614 (2505)	2084 (2005)	2622 (1836)	0.083	0.068	0.030	0.13	0.00
LFn ^9^	63.8 (17.7)	66.4 (16.4)	66.5 (14.7)	71.1 (13.1)	69.6 (14.5)	65.0 (14.7)	0.205	0.516	0.024	0.09	0.01
HF ^10^ [ms^2^]	1785 (2538)	2409 (3568)	1489 (2659)	1548 (1996)	946 (928)	1581 (1388)	0.096	0.012	0.498	0.03	0.00
HFn ^11^	36.2 (17.7)	33.6 (16.4)	33.5 (14.7)	28.9 (13.1)	30.4 (14.5)	35.0 (14.7)	0.205	0.515	0.024	0.09	0.01
LF/HF ^12^	292.3 (314.9)	313.7 (316.9)	293.1 (275.0)	356.8 (361.1)	327.6 (254.6)	276.7 (268.8)	0.323	0.563	0.027	0.09	0.01
Systolic BP ^13^ [mmHg]	127.2 (11.6)	121.3 (11.2)	123.5 (13.2)	119.0 (11.0)	121.6 (13.7)	119.8 (14.7)	0.006*	<0.001 *	0.155	0.07	0.01
Diastolic BP [mmHg]	77.7 (7.6)	78.3 (7.8)	75.8 (7.4)	72.6 (8.1)	73.0 (10.2)	73.5 (8.1)	<0.001*	0.371	0.040	0.00	0.13

^1^ η^2^p: effect size partial η squared, ^2^ BL: baseline, ^3^ FU: follow-up, ^4^ M-C: Outdoor mountain hiking vs. sedentary control condition, ^5^ M-T: Outdoor mountain hiking vs. indoor treadmill walking, ^6^ SDNN: standard deviation of the normal-to-normal beat intervals of heart rate, ^7^ RMSSD: square root of the mean squared differences of successive normal-to-normal beat intervals of heart rate, ^8^ LF: power in low frequency range, ^9^ HF: power in high frequency range, ^10^ LFn: power in low frequency range normalized values, ^11^ HFn: power in high frequency range normalized values, ^12^ LF/HF: ratio between power in low frequency range and power in high frequency range, ^13^ BP: blood pressure, * indicates statistically significant results.
